# Enhancing a Behavioral Intervention Using Rest-Activity Rhythm Monitoring via a Consumer Wearable in Older Dementia Caregivers and People With Dementia: Feasibility and Acceptability Study

**DOI:** 10.2196/88773

**Published:** 2026-09-30

**Authors:** Sarah T Stahl, Juleen Rodakowski, Riddhi Patira, Stephen F Smagula

**Affiliations:** 1 Department of Psychiatry, University of Pittsburgh, 3811 Ohara St, Pittsburgh, PA, 15213-2561, United States, 1 412 246 6003; 2 Department of Occupational Therapy, University of Pittsburgh, Pittsburgh, PA, United States; 3 Department of Neurology, University of Pittsburgh, Pittsburgh, PA, United States

**Keywords:** aging, dementia, caregiver, accelerometer, wearables, Apple Watch, digital mental health, rest-activity rhythm

## Abstract

**Background:**

Despite being established risk factors for poor mental or brain health outcomes in aging, rest-activity rhythm (RAR) disturbances are not routinely monitored or treated. This is, in part, due to a lack of clinician-friendly RAR monitoring systems.

**Objective:**

We tested the feasibility and acceptability of personalizing a 6-week behavioral intervention using RAR monitoring from a consumer wearable device (Apple Watch). We selected a target population study of people with dementia and their family caregivers, given that rest-activity pattern disturbances are common in these groups.

**Methods:**

This single-arm trial enhanced a behavioral activation rhythm treatment with the Apple Watch–based app myRhythmWatch, providing users and their therapists with objective RAR monitoring for customizing therapy. Therapists used information from the myRhythmWatch app to visualize the participants’ behavioral patterns, identify treatment targets, and track progress. Participants included 21 older adults (15 dementia caregivers: mean age 61.8, SD 8.4 years; 6 people with dementia: mean age 81.45, SD 8.5 years). Feasibility outcomes were as follows: (1) proportion adherent enough to assess RARs (defined as ≥3 consecutive valid days with ≥20 hours per day) and (2) the total number of valid days. Acceptability was measured via the Likert scale to gauge participants’ satisfaction with the intervention. We secondarily examined preintervention and postintervention changes in depression (9-item Patient Health Questionnaire scores) and insomnia (Insomnia Severity Index) scores among a smaller group of 11 caregivers who completed these measures.

**Results:**

All 21 participants obtained the minimum data requirement for characterizing an RAR snapshot. Caregivers averaged 35 valid RAR monitoring days, and all 15 caregivers were still using the app at week 6 of the trial. In contrast, people with dementia averaged 30 valid days, and of the 6 people with dementia, only 6 were active users at the end of the trial. On average, caregivers completed 5.7 (SD 0.46) of the 6 therapy sessions offered, and participant satisfaction with program components was “high.” Depression symptoms improved with medium preintervention and postintervention effect sizes (*t*_10_=2.417; *P*=.02; Hedges *g*=0.67, 95% CI 0.04‐1.28), and there were large effect size improvements in insomnia symptoms (*t*_10_=3.377; *P*=.004; Hedges *g*=0.94, 95% CI 0.25‐1.61).

**Conclusions:**

These findings show that older adults without dementia were highly engaged with RAR monitoring. This supports the feasibility of personalizing interventions for older adults with objective RAR monitoring. Randomized controlled trials are warranted to determine whether adding RAR monitoring improves intervention efficiency, efficacy, or durability. While feasible in a subset of people with dementia, we observed lower use rates indicating that there are more barriers to implementing long-term consumer wearable–based RAR monitoring in people with dementia.

## Introduction

Self-monitoring is a major component of several behavioral interventions in psychiatry, and current standard monitoring approaches are based on patient self-reporting [1,2]. For example, in behavioral treatments for insomnia, patients fill out daily sleep diaries [1], and in behavioral activation for depression, logs are used to track activities and reward [2]. Such information theoretically can increase patient self-awareness, formulate cases, guide intervention component selection, and track progress. However, self-report monitoring tools have some notable limitations, namely issues with burden, recall bias, and reliability.

Passive 24-hour sleep/wake rhythm monitoring via a wrist-worn accelerometer provides complementary, objective, and clinically relevant information with less participant burden. Sleep and waking behaviors follow a 24-hour pattern that can be objectively measured via passive wrist accelerometry. Measures extracted from 24-hour accelerometers, often referred to as rest-activity rhythm (RAR) measures [3], quantify several domains of behavior including timing, sleep and activity duration, activity intensity and amplitude at various times of day, sleep and 24-hour fragmentation, and circadian regularity. A large and growing evidence base indicates that, especially when various aspects of RAR disruption co-occur, people are more likely to develop depression symptoms [4,5], cardiometabolic disease [6,7], dementia [8-10], worse cancer treatment outcomes [11,12], and even earlier mortality [13,14]. As RAR measures capture behaviors that are susceptible to voluntary or exogenous control, health-relevant aspects of RAR disruption are often considered modifiable intervention targets.

We proposed that monitoring RARs with consumer-based wearable accelerometer devices can provide a scalable approach to improve the efficiency and precision of behavioral interventions [15]. Consumer wearable devices are already widely used and provide logical platforms for multiple “digiceuticals” or clinical applications of digital technology, such as the clinical uses of RAR monitoring. We previously demonstrated the feasibility of real-time RAR monitoring using raw accelerometer data from the Apple Watch [15]. This Apple Watch–based system, called myRhythmWatch, produces RAR measures that are largely consistent in rank order with those obtained using standard research-based wrist accelerometers [16]. Relatively short-term use, for example, a week, of myRhythmWatch is feasible in older adults with and without mild cognitive impairment, and the resulting RAR measures correlated with cognitive performance [17].

To the best of the authors’ knowledge, no prior studies have evaluated if it is feasible to tailor behavioral interventions using ongoing sleep, wake, or RAR monitoring via wearables. Two past studies used daytime wearable data, for example, step counts, to tailor exercise interventions [18,19]. Another prior study gave patients Oura Smart Rings to self-monitor their sleep during digital cognitive behavioral therapy for insomnia (refer to the study by Moon et al [20]; this study used a sleep diary and did not use wearable sensing data to tailor the intervention). There are several key limitations of these prior studies. First, they relied on measures calculated via proprietary algorithms from consumer wearable companies, which may introduce replication problems if algorithms change. Second, past studies in this area have focused on sleep and physical activity, but none have focused on 24-hour patterns or RARs, which have unique health relevance (15-17). An existing mobile sensing app, called Rhythm, produces rhythm measures [21]. However, metrics from the Rhythm app are derived based on smartphone use instances (ie, not from wrist accelerometry); therefore, they are fundamentally incompatible with the accelerometer-based RAR measures used in health research. Third, and most critically, we are unaware of existing wearable-based systems featuring a “Clinician Dashboard” user interface that delivers information from wearables to providers in real time to facilitate intervention tailoring.

To fill these gaps, our primary aim was to evaluate the feasibility and acceptability of enhancing a standardized behavioral intervention with RAR monitoring via the myRhythmWatch system. For this study, we selected a target population of people with dementia and their older adult caregivers who are family members. People with dementia more frequently experience RAR disruption when compared with healthy controls [22,23]. Several prior intervention studies have targeted rhythm disruption in people with dementia [24-29]; therefore, people with dementia represent one end-user subgroup for testing whether enhancing interventions with RAR monitoring is feasible and acceptable. Objective rhythm monitoring may be especially important in people with dementia, given that cognitive impairment may limit the reliability of subjective reports in people with dementia. Distinct alterations to RAR patterns are also common in caregivers of people with dementia (eg, due to caregiving and related psychological stress) [30-33]; thus, dementia caregivers are another group in whom rhythm monitoring or interventions are needed. However, as noted above, the feasibility of enhancing interventions using consumer-based RAR monitoring in any group (let alone groups with dementia or experiencing caregiving stress) has yet to be established.

Therefore, our primary aim was to evaluate the feasibility and acceptability of enhancing a rhythm-focused behavioral intervention using RAR monitoring in people with dementia and their caregivers. Circadian interventions and RAR monitoring were not designed specifically for people with dementia and dementia caregivers. However, due to the high prevalence of rhythm disruption in people with dementia and dementia caregivers, these subgroups represent more plausible and important real-world end users than unselected healthy or normative samples. We secondarily evaluated preintervention and postintervention changes in depression and insomnia symptoms.

## Methods

### Participants

Participants enrolled as caregiver singletons or caregiver–care recipient dyads. Eligibility criteria for caregivers were as follows: (1) aged >50 years; (2) is a primary caregiver for a patient with Alzheimer disease (AD) or an AD-related dementia; (3) experiencing stress or strain related to caregiving; (4) the person being cared for had problems with sleep or keeping a consistent routine; (5) experiencing at least mild depression symptoms defined as a score ≥5 on the 9-item Patient Health Questionnaire (PHQ-9); (6) living with their care recipient; and (7) willing to try the app and participate in assessments and calls. Eligibility criteria for care recipients were as follows: (1) aged >60 years; (2) having been diagnosed with AD, vascular dementia, mixed Alzheimer or vascular dementia, or frontotemporal dementia; (3) living with their caregiver; and (4) willing to wear the watch and participate in assessments and calls.

Enrollment and follow-up procedures are presented in Multimedia Appendix 1 [34]. There were 25 caregivers screened, of which 3 did not meet the inclusion criteria and 7 were not interested, leaving 15 eligible or interested caregivers. Note that the requirement of at least mild depression symptoms was added after the study began, based on stakeholder feedback that the program worked or was most appropriate for caregivers with at least some depression symptoms. This modified inclusion criteria allowed us to identify and enroll more caregiver–care recipient dyads. Caregivers enrolled after the change in PHQ-9 eligibility (n=3) reported more depressive symptoms (mean 8.83, SD 5.81) compared to caregivers enrolled before the change (n=12; mean 5.69, SD 5.28). We invited the 15 interested and eligible caregivers to involve their care recipient. A total of 7 of these said they were not interested or did not think the study would be appropriate for their care recipient, and 2 care recipients were ineligible, rendering a sample of 6 care recipients (people with dementia).

### Ethical Considerations

The University of Pittsburgh Institutional Review Board approved all procedures (STUDY21060122), and the trial was registered with ClinicalTrials.gov (NCT05309577). Participants provided informed electronic consent (e-consent) in REDCap (Vanderbilt University). To include individuals representative of the population of people with dementia, we allowed individuals who did not have the capacity to give informed consent to enroll, provided they gave verbal assent and proxy consent signed by a legally authorized representative. Proxy consent was described as the process of honoring the care recipient’s values and making medical decisions on their behalf when they can no longer do so themselves. Of the 6 care recipients (people with dementia), 3 (50%) signed their own e-consent in REDCap; the remaining 3 (50%) provided consent by proxy, and their caregiver consented on their behalf. All data were deidentified. The watches and the myRhythmWatch app did not record any identifiable data; they recorded only movement and acceleration intensity. Participants were compensated US $125 for completing study procedures.

### Procedures

This single-arm pilot trial enrolled participants between March 2023 and April 2024 in Pittsburgh, United States. Participants were recruited from a University of Pittsburgh research registry (Pitt+Me [35]). The registry includes more than 400,000 participants who agreed to be considered for research studies at the University of Pittsburgh. Targeted emails describing this clinical trial were sent to members who identified as adult caregivers. The study coordinator would receive a list of interested members, and we completed the screening and consent during the same initial phone call. Self-report and clinical health assessments were conducted with nonblinded research staff at 3 time points: baseline, 6 weeks after the intervention, and 3 months after the intervention (caregivers only). All assessments were completed over the phone or virtually. The completed TREND (Transparent Reporting of Evaluations with Nonrandomized Designs) checklist is provided in Checklist 1 [36].

After enrollment, all 21 participants (caregiver singletons and caregiver–care recipient dyads) received one-on-one orientation to wearing the Apple Watch and using the myRhythmWatch app. Of the 21 participants, 18 (86%) preferred virtual orientation; the remaining 3 (14%) requested an in-person orientation. Participants were provided an Apple Watch Series 8 via the United States Postal Service and asked to wear it over the 6-week trial, except when they were bathing or showering, when we asked them to charge the watch. As needed, we also followed up with participants 2 days after the technology orientation to troubleshoot any issues that arose. Of the 15 caregivers, 11 (73%) also completed pretreatment and posttreatment assessments and were included in the health outcome analysis. Incomplete assessments (4/15, 27% caregivers) were linked to scheduling conflicts and low caregiver availability. There were no statistical differences in age, mood, or insomnia between caregivers who did and did not complete the assessments. However, these findings should be interpreted with caution given the low sample size (Multimedia Appendix 2).

### Intervention

The myRhythmWatch system uses raw accelerometry data from the Apple Watch, as previously described [15-17], to compute standard RAR metrics that are displayed to users on their mobile phones (Figure 1). For technical details on handling raw accelerometer data, refer to the study by Zhang et al [16]. Briefly, the raw triaxial accelerometer data were bandpass filtered, aggregated across axes, and then converted to 30-second epochs. Standard RAR measures are then extracted and presented as standardized scores. Therapists accessed information on participants’ RARs via a Health Insurance Portability and Accountability Act (HIPAA)–compliant clinician dashboard, which displayed participants’ RAR metric data (Figure 2) and graphs including raw accelerometer data plots (Figure 3). Therapists used this information in 6 sessions, 1 per week, each lasting about 30 to 45 minutes. Caregivers facilitated sessions for themselves and for their care recipients; when care recipients were involved, sessions lasted approximately 60 minutes. All 21 participants (caregiver singletons and caregiver–care recipient dyads) received the 6-week intervention.

In the first session, and as needed thereafter, therapists reviewed standardized educational materials about circadian rhythms and health. They also used open-ended questions to understand the relevance of rhythms to the participant’s life or health goals. The rest of session 1, and all subsequent sessions, focused on reviewing findings from the myRhythmWatch app and used behavioral activation techniques to plan activities focused on supporting a healthy rhythm [37]. There was an explicit focus on adhering to 4 Rules for Healthy Rhythms: (1) waking up at the same time every day, (2) engaging in rewarding morning activity (preferably with exposure to sunlight and physical activity), (3) limiting naps to <30 minutes and ending before 4 PM, and (4) maintaining active periods of around 15 to 16 hours per day.

**Figure 1.**
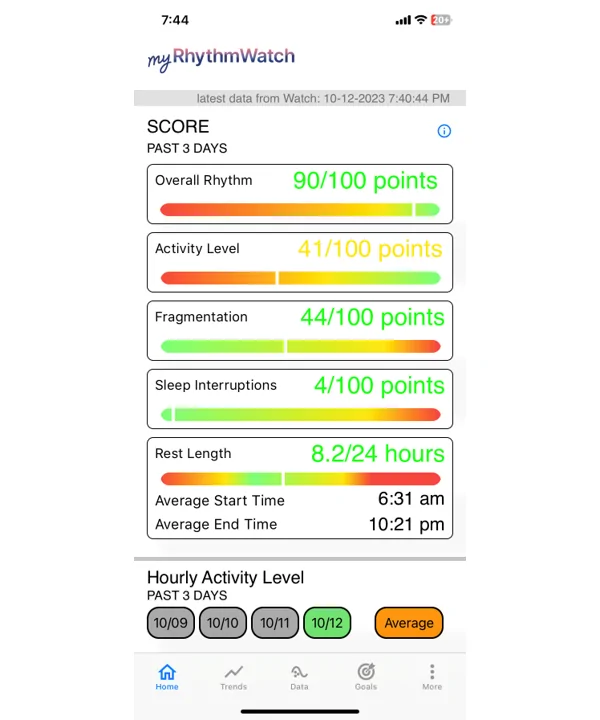
User interface of the myRhythmWatch app. The mobile phone interface displayed rest-activity rhythm scores using color bar graphics.

**Figure 2.**
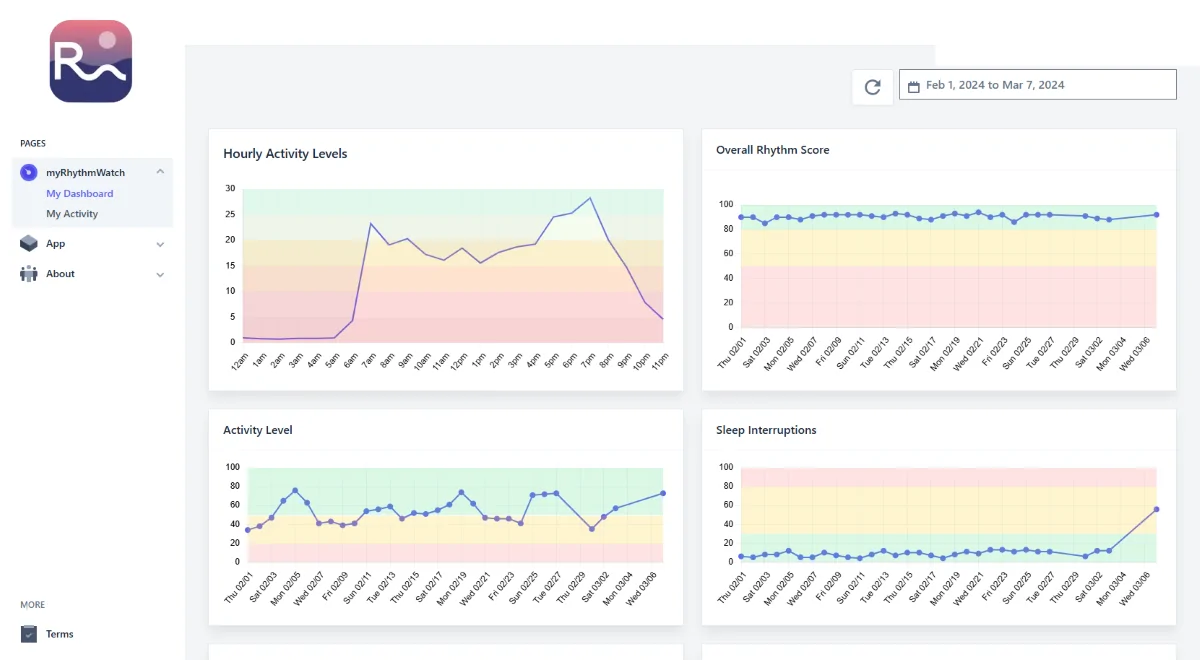
User interface of the myRhythmWatch app: the clinician dashboard displayed average profiles over a selected period and rest-activity rhythm measure data.

**Figure 3.**
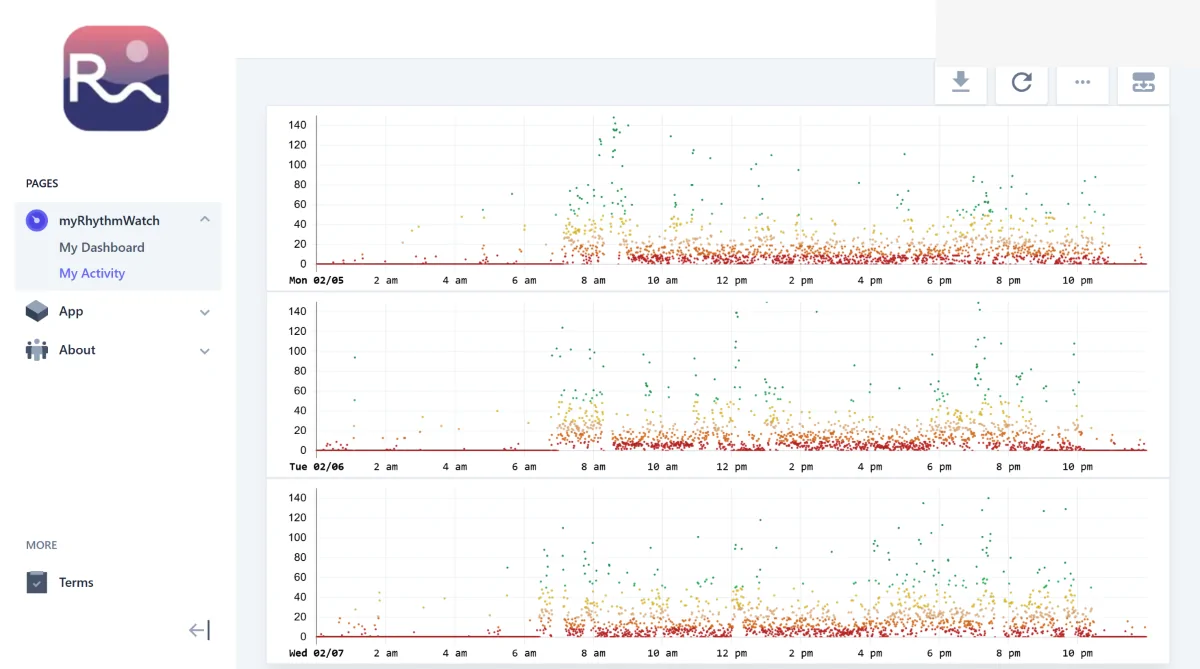
User interface of the myRhythmWatch app: clinician dashboard page showing raw accelerometer data plots.

### Feasibility and Acceptability Measures

Feasibility was assessed with process outcomes including (1) the proportion of users providing sufficient data for assessing RARs (ie, ≥3 consecutive valid days defined as days with at least 20 hours of data) and (2) the total number of valid days of accelerometer data collected. Program acceptability was measured using the total number of completed health coaching visits. Participants also rated satisfaction with intervention components on a 5-point Likert scale, ranging from “very dissatisfied” (score=1) to “very satisfied” (score=5). Scores ≥4 indicate adequate levels of intervention acceptability.

### Mood and Sleep Measures

Initially, the intervention was designed to focus on promoting self-care and reducing caregiving burden. However, in designing the trial, the focus was switched to the circadian-informed, behavioral activation–based approach (described above) that aimed to improve mood and sleep in caregivers. Mood and sleep measures were retained as secondary outcomes in the final trial that included (1) depression symptom severity assessed using the PHQ-9 [38] (scored on a 4-point Likert scale [scores=0-3] and summed to a maximum severity score of 27) and (2) insomnia symptom severity assessed using the Insomnia Severity Index (ISI [39]; scored on a 4-point Likert scale [scores=0-3] and summed to a maximum severity score of 28). The measures were chosen to be consistent with prior research on mood, sleep, and circadian rhythms in dementia caregivers [40].

Secondary outcome measures of mood and sleep were also included. Depression was measured with the 20-item Center for Epidemiological Studies Depression Scale [41]. Item responses range from 0 (rarely or none of the time) to 3 (most of the time), with a scale range of 0 to 60 (higher scores reflect increased depressive symptom severity). Anxiety symptoms were assessed using the 7-item Generalized Anxiety Disorder Scale [42]. Item responses range from 0 (none of the time) to 3 (most of the time), with a scale range of 0 to 21 (higher scores reflect increased anxiety symptom severity). Measures of sleep/circadian functioning were also included. Sleep quality was assessed using the 19-item Pittsburgh Sleep Quality Index [43]. Item responses range from 0 (no difficulty or problem) to 3 (severe difficulty) with a scale range of 0 to 21 (higher scores indicate worse sleep quality). Daytime sleepiness was assessed using the 8-item Epworth Sleepiness Scale [44]. Item responses range from 0 (would never doze) to 3 (high chance of dozing) with a scale range of 0 to 24 (higher scores indicate more excessive daytime sleepiness). Circadian preference was assessed using the 13-item Composite Scale of Morningness [45]. Item responses are time increments that range from 1 to 4 or 5 with a total scale range of 13 to 55 (higher scores indicate a greater degree of morningness).

### Statistical Analyses

Feasibility and acceptability outcomes were analyzed descriptively for caregivers and care recipients (people with dementia). We used paired-sample 2-tailed *t* tests to report preintervention and postintervention change scores in caregivers’ mood and sleep over 6 weeks. Hedges *g* effect sizes were calculated and interpreted according to established standards (0.3 indicates a small effect, 0.5 indicates a medium effect, and >0.8 indicates a large effect). Caregivers with incomplete assessments (n=4) were excluded from the pre-post analyses. Our sample size was determined (approximately 20 participants) to ensure it met practical resources and timeline constraints for determining feasibility and acceptability. All analyses were conducted using SPSS (version 31; IBM Corp).

## Results

### Overview

Consistent with the known gender distribution of dementia caregivers [46], caregiver participants were mostly women (12/15, 80%) providing for a loved one with dementia (9 caregiver singletons and 6 caregiver–care recipient dyads). In total, 24% (4/15) of participants were Black or African American (Table 1). Of the 21 participants (caregiver singletons and caregiver–care recipient dyads) enrolled in the study, all of them completed the myRhythmWatch intervention.

**Table 1.** Participant characteristics at baseline.^a^

	Caregivers (n=15)	Care recipients (people with dementia; n=6)
Sociodemographics		
Age (years), mean (SD)	61.78 (8.4)	81.45 (8.46)
Sex, n (%)		
Female	13 (87%)	4 (66.7)
Male	2 (13%)	2 (33.3)
Race, n (%)		
Black	4 (26.7)	1 (16.7)
White	10 (66.6)	5 (83.3)
Did not report	1 (6.7)	—^b^
Education, n (%)		
High school diploma	2 (13.3)	—
Some college	3 (20.0)	—
College degree	3 (20.0)	—
Some postgraduate college	1 (6.7)	—
Postgraduate college	6 (40.0)	—
Medical illness burden (CIRS-G^c^), mean (SD)	7.73 (6.55)	—
Mood measures, mean (SD)		
Depressed mood (PHQ-9^d^)	6.64 (5.00)	—
Depressed mood (CESD-R^e^)	13.07 (9.86)	—
Anxiety (GAD-7^f^)	4.57 (4.38)	—
Sleep measures, mean (SD)		
Insomnia (ISI^g^)	11.43 (5.10)	—
Circadian preference (CSM^h^)	28.43 (6.65)	—
Sleep quality (PSQI^i^)	8.79 (3.68)	—
Daytime sleepiness (ESS^j^)	7.14 (4.90)	—

a No adverse events were reported during the pilot study period.

b Not applicable.

c CIRS-G: Cumulative Illness Rating Scale for Geriatrics (score range 0‐56).

d PHQ-9: 9-item Patient Health Questionnaire (score range 0‐27).

e CESD-R: Center for Epidemiologic Studies Depression-Revised (score range 0‐60).

f GAD-7: 7-item Generalized Anxiety Disorder (score range 0‐21).

g ISI: Insomnia Severity Index (score range 0‐28).

h CSM: Composite Scale of Morningness (score range 0‐55).

i PSQI: Pittsburgh Sleep Quality Index (score range 0‐21).

j ESS: Epworth Sleepiness Scale (score range 0‐24).

### Primary Outcomes: Feasibility and Acceptability

#### Feasibility of App Use and RAR Data Quality

All participants, in both caregivers and care recipients, achieved the minimum requirement to extract at least 1 RAR measurement (ie, at least 3 consecutive valid days). The total number of valid accelerometer days was 35.5 (SD 2.7) in caregivers and 30.0 (SD 3.2) in care recipients. For caregivers, a few participants were under the minimum data collection requirement during the first week (Figure 4). In subsequent weeks, on average, caregivers collected sufficient daily data, and all caregivers were still actively collecting data on the app during the last week of the trial. In contrast, among care recipients, average data collection rates were lower and more variable throughout the trial (Figure 5). Because of increasing data collection issues among people with dementia, the study was modified to ask caregivers to use the app with their people with dementia for only 1 month.

**Figure 4.**
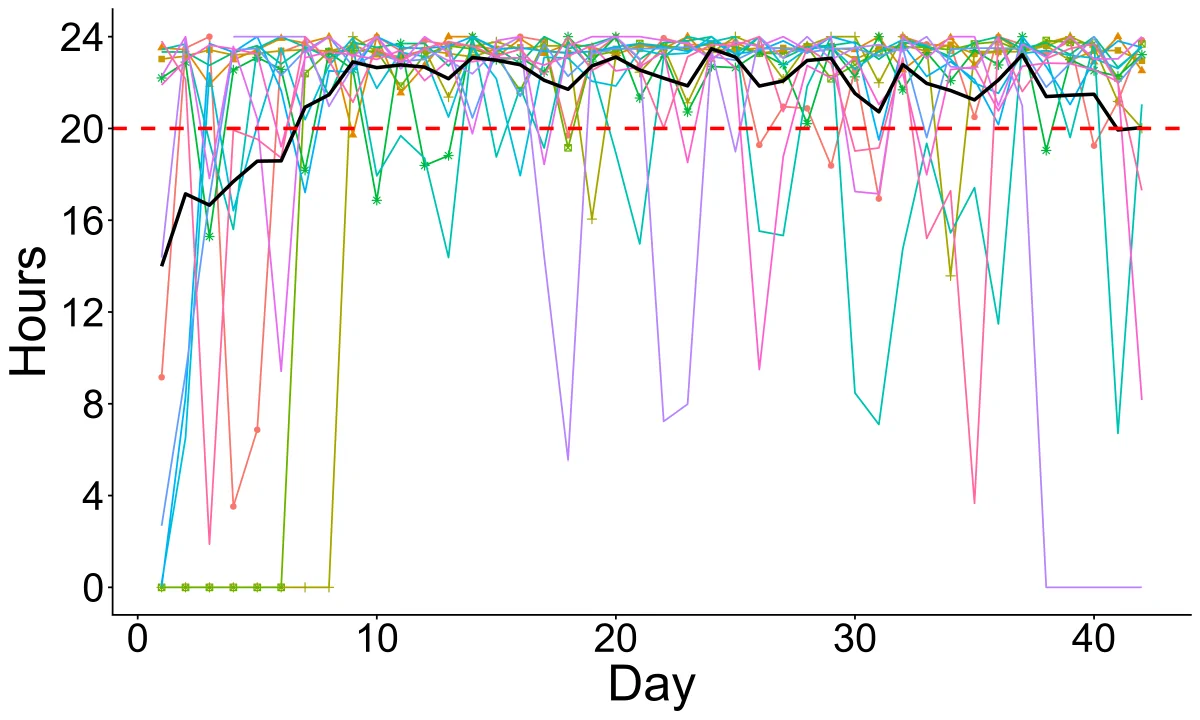
Number of hours of accelerometer data obtained per day from 15 dementia caregivers. Each line represents 1 study participant; the black line represents the mean number of hours obtained per day.

**Figure 5.**
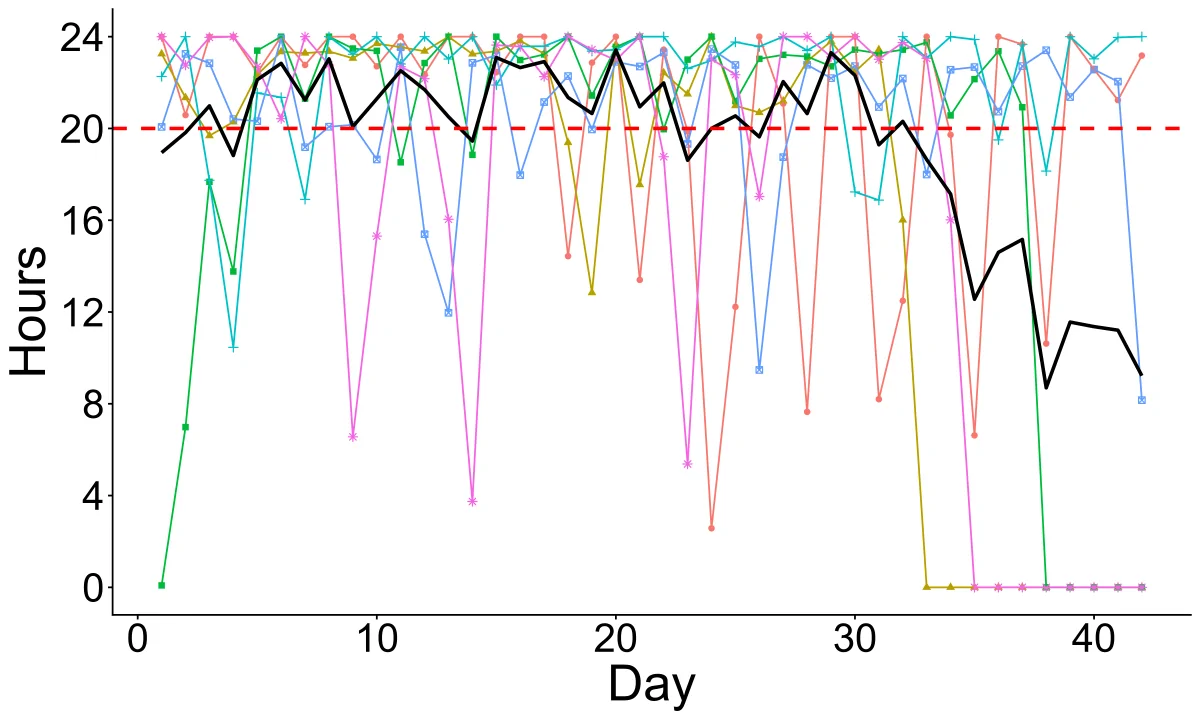
Number of hours of accelerometer data obtained per day from 6 care recipients (people with dementia). Each line represents 1 study participant; the black line represents the mean number of hours obtained per day.

#### Acceptability

All participants attended 5 (27%) or 6 (73%) of the 6 therapy sessions offered. Participants rated high intervention acceptability (mean 4.8, SD 0.44; observed range 4-5). Acceptability of intervention components, based on written feedback, was also high.

### Secondary Outcomes: Preintervention and Postintervention Changes in Mood and Sleep

Hedges *g* effect size estimates indicate medium to large effect size changes for all outcomes except for anxiety symptom severity (Table 2). Total PHQ-9 scores were reduced by an average of 58% from baseline to week 6. ISI scores were also reduced by an average of 58% from baseline to week 6. Statistically significant improvements were observed in multiple dimensions of sleep including improved sleep quality, less daytime sleepiness, and an increased circadian preference for morningness. To further illustrate key changes observed, we plotted individual and group preintervention and postintervention PHQ-9 (Figure 6) and ISI scores (Figure 7).

**Table 2.** Hedges *g* effect size differences in mood and sleep over 6 weeks in dementia caregivers (n=11).

	Preintervention mean (SD)	Postintervention mean (SD)	*t* test (*df*)	Hedges *g* (95% CI)	*P* value
Mood measures					
Depressed mood (PHQ-9^a^^,^^b^)	5.82 (4.79)	2.27 (2.61)	2.477 (10)	0.67 (0.042 to 1.278)	.02
Depressed mood (CES-D^c^)	11.18 (7.51)	6.09 (6.64)	2.239 (10)	0.62 (0.002 to 1.219)	.03
Anxiety (GAD-7^d^)	4.36 (4.86)	2.45 (2.84)	1.259 (10)	0.35 (–0.224 to 0.908)	.12
Sleep measures					
Insomnia (ISI^a^^,^^e^)	10.91 (5.68)	5.64 (5.73)	3.377 (10)	0.94 (0.245 to 1.605)	.004
Sleep quality (PSQI^f^)	8.00 (3.63)	6.18 (2.96)	5.186 (10)	0.84 (0.173 to 1.484)	.006
Circadian preference (CSM^g^)	30.18 (6.27)	34.45 (7.08)	–2.246 (10)	–0.63 (–1.221 to –0.004)	.02
Daytime sleepiness (ESS^h^)	7.73 (5.30)	5.00 (3.03)	2.659 (10)	0.73 (0.088 to 1.349)	.01

a Primary mood and sleep outcomes of interest.

b PHQ-9: 9-item Patient Health Questionnaire.

c CES-D: Center for Epidemiologic Studies-Depression.

d GAD-7: 7-item Generalized Anxiety Disorder.

e ISI: Insomnia Severity Index.

f PSQI: Pittsburgh Sleep Quality Index.

g CSM: Composite Scale of Morningness.

h ESS: Epworth Sleepiness Scale.

**Figure 6.**
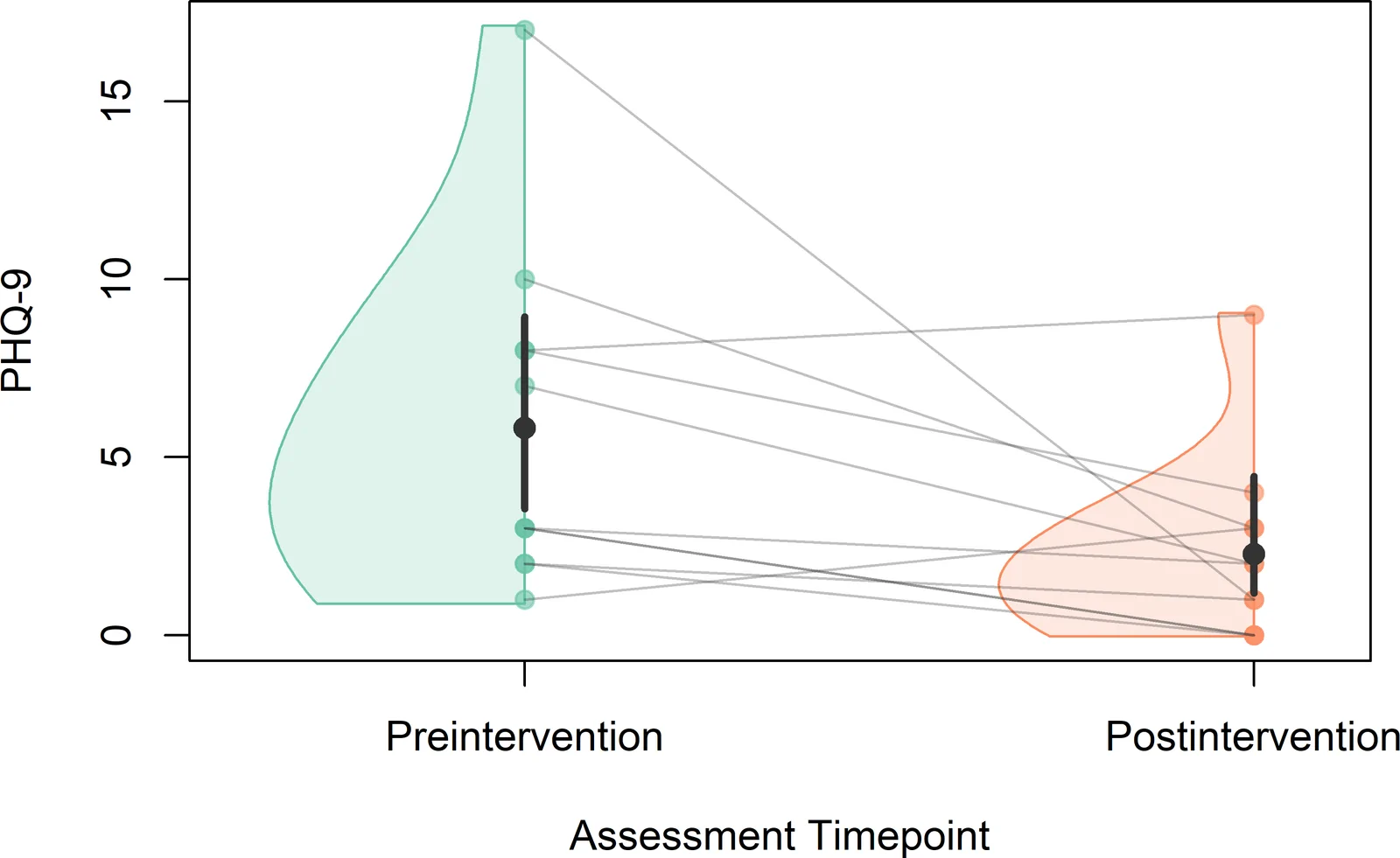
Preintervention and postintervention changes in 11 dementia caregivers: individual and group preintervention and postintervention depressed mood scores (9-item Patient Health Questionnaire [PHQ-9]).

**Figure 7.**
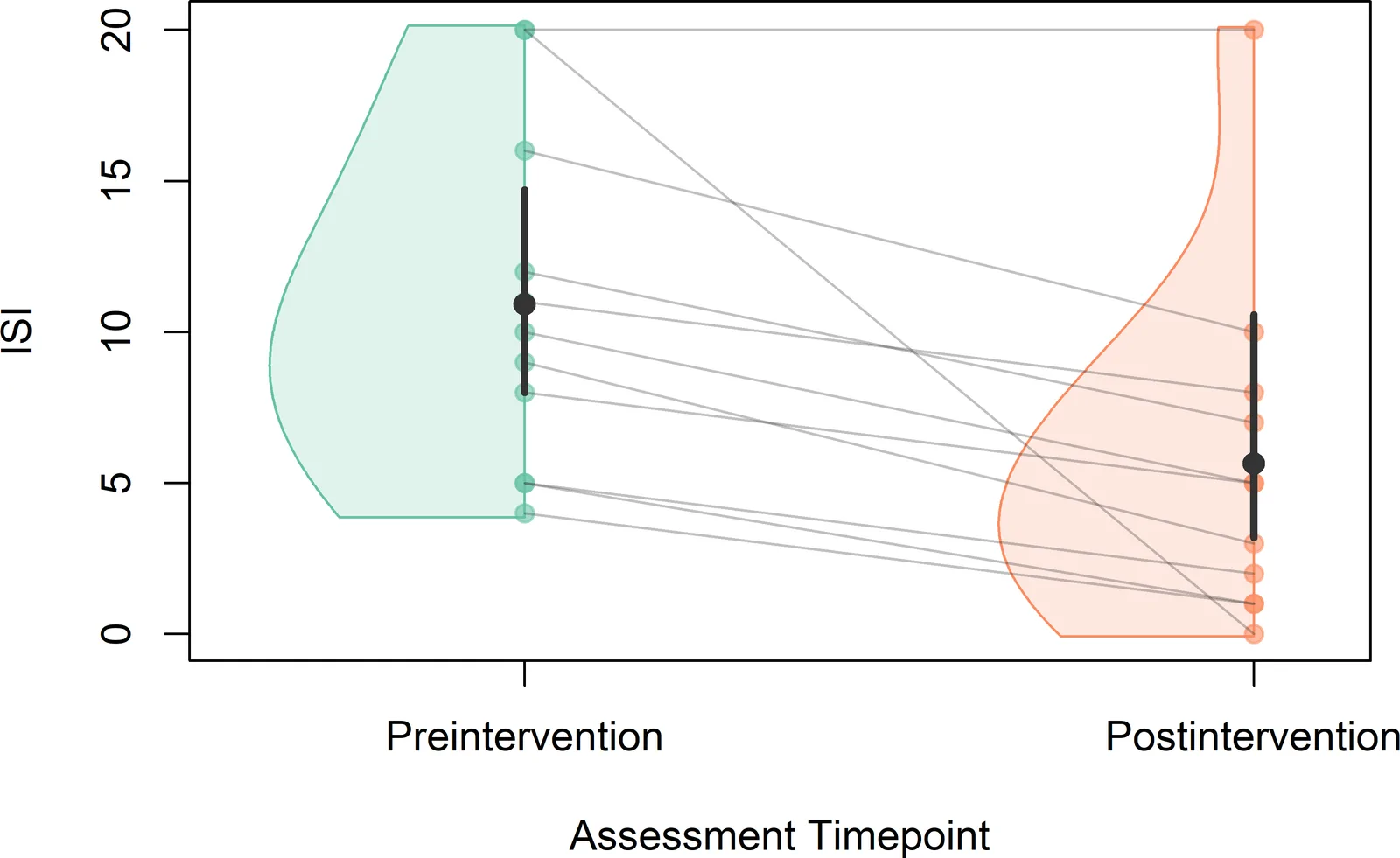
Preintervention and postintervention changes in 11 dementia caregivers: individual and group preintervention and postintervention insomnia scores (Insomnia Severity Index [ISI]).

## Discussion

### Principal Findings

Overall, findings suggest that personalizing a behavioral intervention using consumer-based RAR monitoring is feasible and acceptable in older adults experiencing stress related to their role as dementia caregivers. This conclusion is supported by the high and sustained engagement with the myRhythmWatch app observed among caregivers in this sample. There were also significant preintervention and postintervention improvements in mental health, with dementia caregivers reporting fewer depression symptoms, less insomnia, less daytime sleepiness, and more “morningness” at the end of the trial.

Another novel finding is that RAR monitoring using a consumer wearable was feasible in people with dementia, albeit with significant caveats. First, most caregivers (9/15, 60%) did not think the study would be appropriate for their care recipients who had dementia. Second, among the 6 people with dementia who volunteered for the study, engagement was lower and for a shorter term compared with that among caregivers. Thus, it is feasible to use the Apple Watch to perform RAR assessments in people with dementia but not in all patients and typically not over longer periods.

### Comparison to Prior Work

Most prior research using accelerometers has used recordings, over periods of 1 to 2 weeks, as a measurement tool in association studies [4,5]. A few prior studies have demonstrated success measuring RARs using raw accelerometer data from the Apple Watch [15-17]. The present study is novel in that it is the first to demonstrate the feasibility of using this approach for (1) longer-term RAR monitoring and (2) tailoring the delivery of a behavioral intervention. Our findings are also novel in that the myRhythmWatch system’s clinician dashboard provides the first real-world or scalable approach to monitor measures of RAR disturbances in clinical practice.

The approach here also differs notably from prior mobile phone app–based research in this area. As noted in the Introduction section, similar apps have relied on phone use data [21] or step counts [18,19]. An advantage of using raw epoch-by-epoch accelerometer data here is that this allows us to characterize 24-hour behavioral patterns using the same RAR measures that have established health relevance in research [5,13,37]. We observed consistent engagement with the myRhythmWatch app for more than 6 weeks, which compares favorably with other mobile health apps. While approximately 40% of people drop out of mobile health interventions [47,48], all participants in the present study were retained, and caregivers remained engaged with the app after 6 weeks.

Prior studies have shown that people with dementia tend to have more fragmented and unstable RARs [22,23,30]. These results raise the possibility that myRhythmWatch may be useful for long-term monitoring in subgroups of patients with dementia. However, given the relatively lower engagement among people with dementia, future studies should consider integrating even less obtrusive measurement approaches, such as a wearable pin or pendant, as used by Figueiro et al [49]. In contrast, despite the stressors of being a dementia caregiver, nearly all caregivers were consistently able to use this Apple Watch–based system. This suggests that myRhythmWatch may be useful for long-term monitoring for RAR disruptions that are associated with poor health outcomes in caregivers [30-33].

### Limitations

Some limitations and caveats to our interpretations should be noted. First, due to cost restraints and the early-phase nature of this study, we chose a single-arm design to maximize the number of trial participants addressing our primary aims (eg, acceptability of enhancing a behavioral intervention with myRhythmWatch). However, this precludes inference on the extent to which the observed health benefits were due to personalization via real-time RAR monitoring, rather than other aspects of the intervention, nonspecific therapeutic effects, or spontaneous improvements. Second, because our early participants had low or no depressive symptoms, we adjusted our inclusion criteria to include those with at least mild depressive symptoms so that we could study feasibility and acceptability in those experiencing caregiving stress. Third, our sample consisted of older dementia caregivers and people with dementia who were willing or able to use digital technologies. Our findings only speak to the feasibility and acceptability of the myRhythmWatch app among these older adults under caregiving stress or with a serious medical condition (dementia), and results will not necessarily generalize to other subgroups in the broader population. The feasibility and acceptability of the myRhythmWatch app is likely lower among individuals with lower digital literacy. Because the sample included older dementia caregivers, who tend to be predominantly female, future studies in samples with more older men are also warranted [46]. Separate studies will also be needed to confirm feasibility or acceptability in younger people. Finally, due to the small sample size, effect size estimates should be replicated in larger studies.

### Implications and Conclusions

As discussed above, we have shown that personalizing interventions via real-time RAR monitoring is feasible and acceptable in older dementia caregivers and people with dementia. The main immediate implication is that future large-scale randomized controlled trials, using the myRhythmWatch system to enhance behavioral interventions, are feasible in studies with similar older adult target populations. Personalizing treatments based on RAR monitoring is theoretically relevant not only for enhancing the rhythm-focused program used here but also for several behavioral approaches that depend on monitoring activity patterns such as behavioral activation [50], interpersonal and social rhythm therapy [51], and behavioral sleep interventions [52]. Given the feasibility and acceptability demonstrated in subgroups of older adults in this pilot study, future controlled studies are warranted to determine whether adding RAR monitoring to behavioral interventions improves their efficiency, efficacy, or durability of effect.

## Supplementary material

10.2196/88773 Multimedia Appendix 1 CONSORT chart for the pilot myRhythmWatch trial.

10.2196/88773 Multimedia Appendix 2 Characteristics of caregivers who did (n=11) and did not (n=4) complete the pretreatment and posttreatment outcome assessments.

10.2196/88773 Checklist 1 TREND checklist.

## Abbreviations

AD: Alzheimer disease
e-consent: electronic consent
HIPAA: Health Insurance Portability and Accountability Act
ISI: Insomnia Severity Index
PHQ-9: 9-item Patient Health Questionnaire
RAR: rest-activity rhythm
TREND: Transparent Reporting of Evaluations with Nonrandomized Designs
