# Supplementary material for: Enhancing a Behavioral Intervention Using Rest-Activity Rhythm Monitoring via a Consumer Wearable in Older Dementia Caregivers and People With Dementia: Feasibility and Acceptability Study
Source: JMIR Form Res. 2026 Sep 30;10:e88773. doi: 10.2196/88773 (PMC13626640; doi:10.2196/88773)

Figure 1. CONSORT chart for pilot myRhythmWatch trial

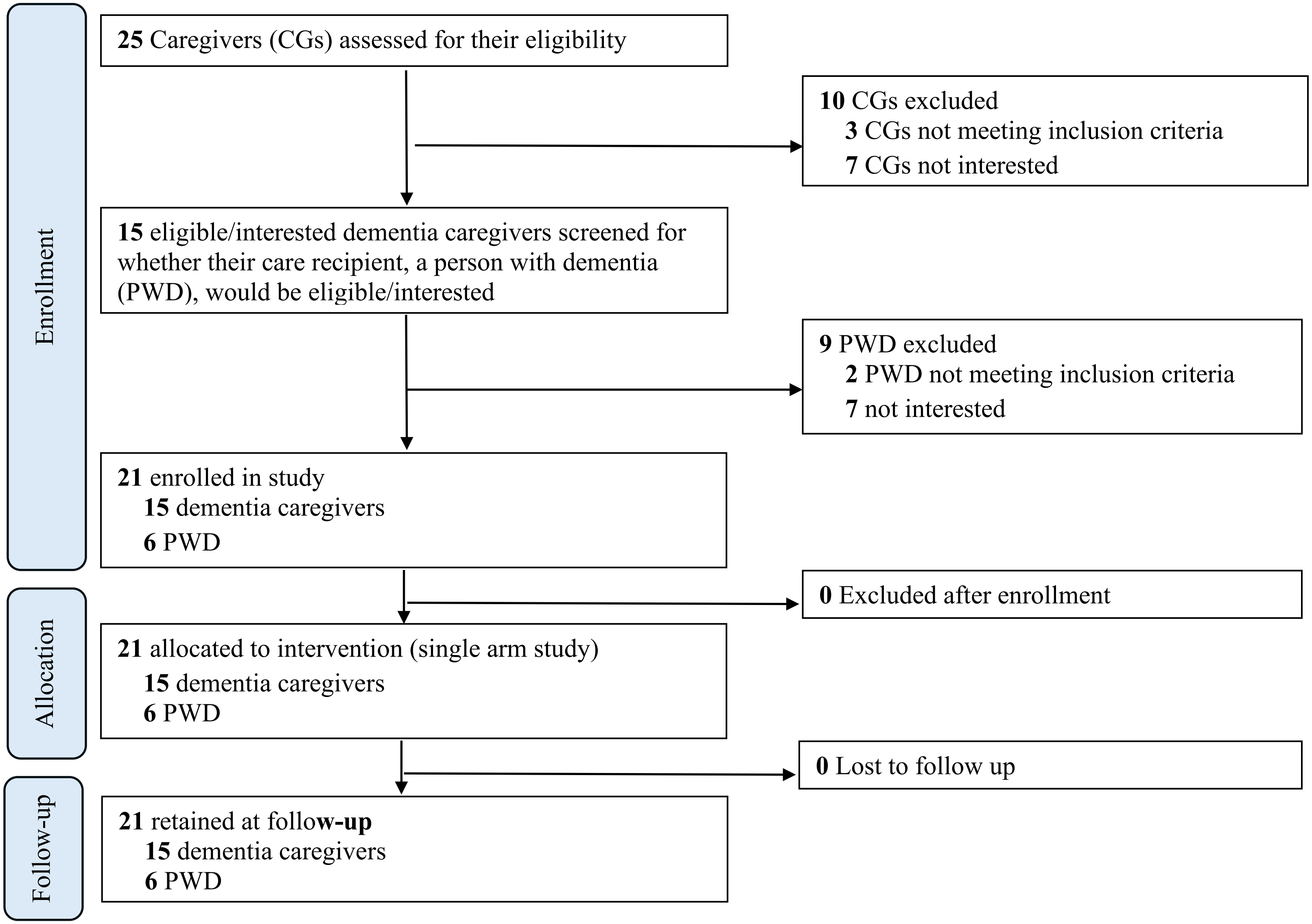

Supplement: Supplementary file 1 [file formative-v10-e88773-s001.pdf]
