# Supplementary material for: Enhancing a Behavioral Intervention Using Rest-Activity Rhythm Monitoring via a Consumer Wearable in Older Dementia Caregivers and People With Dementia: Feasibility and Acceptability Study
Source: JMIR Form Res. 2026 Sep 30;10:e88773. doi: 10.2196/88773 (PMC13626640; doi:10.2196/88773)
Supplement: Supplementary file 2 [file formative-v10-e88773-s002.pdf]

Supplemental Table 1. Characteristics of caregivers who did (11) and did not (4) complete the pre-post treatment outcome assessments

	Caregivers with complete data (n=11)	Caregivers with incomplete data (n=4)	P-Value
Sociodemographics			
Age, y (M (SD)	63.27 (8.76)	59.78 (7.76)	0.49
Sex, n (%)			0.48
Female	10 (90.9)	3 (75.0)	
Male	1 (9.1)	1 (25.0)	
Race, n (%)			0.26
White	6 (54.5)	4 (100.0)	
Black	4 (36.4)	0 (0.0)	
Did not report	1 (9.1)	0 (0.0)	
Education, n (%)			0.23
High school diploma	1 (9.1)	1 (25.0)	
Some college	2 (18.2)	1 (25.0)	
College degree	1 (9.1)	2 (50.0)	
Some post graduate college	1 (9.1)	0 (0.0)	
Post graduate college	6 (54.5)	0 (0.0)	
Medical illness burden, CIRS-G, M (SD)	6.27 (4.38)	11.75 (10.34)	0.16
Mood measures			
Depressed mood, PHQ-9, M (SD)	5.82 (4.79)	9.67 (5.51)	0.25
Depressed mood, CESD-R, M (SD)	11.18 (7.51)	20.00 (16.09)	0.18
Anxiety, GAD-7, M (SD)	4.36 (4.86)	5.33 (2.31)	0.75
Sleep measures			
Insomnia, ISI, M (SD)	10.91 (13.33)	13.33 (0.58)	0.49
Circadian preference, CSM, M (SD)	30.18 (6.27)	22.00 (3.46)	0.06
Sleep quality, PSQI, M (SD)	8.00 (3.68)	11.67 (2.52)	0.13
Daytime sleepiness, ESS, M(SD)	7.73 (5.29)	5.00 (2.65)	0.41

Notes. Data are presented as M (SD) or n (%). Abbreviations: CIRS-G: Cumulative Illness Rating Scale for Geriatrics (score range = 0-56); PHQ-9: Patient Health Questionnaire (score range = 0-27); CESD: Center for Epidemiologic Studies-Depression Revised (score range = 0-60); GAD-7: Generalized Anxiety Disorder – 7 (score range = 0-21); ESS: Epworth Sleepiness Scale (score range = 0-24); CSM: Composite Scale of Morningness (score range = 0-55); PSQI: Pittsburgh Sleep Quality Index (score range = 0-21); and ISI: Insomnia Severity Index (score range 0-28).
